# Administration of hydrogen sulfide via extracorporeal membrane lung ventilation in sheep with partial cardiopulmonary bypass perfusion: a proof of concept study on metabolic and vasomotor effects

**DOI:** 10.1186/cc10016

**Published:** 2011-02-07

**Authors:** Matthias Derwall, Roland CE Francis, Kotaro Kida, Masahiko Bougaki, Ettore Crimi, Christophe Adrie, Warren M Zapol, Fumito Ichinose

**Affiliations:** 1 Anesthesia Center for Critical Care Research, Department of Anesthesia, Critical Care and Pain Medicine, Massachusetts General Hospital and Harvard Medical School, 55 Fruit Street, Boston, MA 02114, USA; 2 Department of Anesthesia, Medical Faculty, RWTH Aachen University, Pauwelsstrasse 30, D-52074 Aachen, Germany

## Abstract

**Introduction:**

Although inhalation of 80 parts per million (ppm) of hydrogen sulfide (H_2_S) reduces metabolism in mice, doses higher than 200 ppm of H_2_S were required to depress metabolism in rats. We therefore hypothesized that higher concentrations of H_2_S are required to reduce metabolism in larger mammals and humans. To avoid the potential pulmonary toxicity of H_2_S inhalation at high concentrations, we investigated whether administering H_2_S via ventilation of an extracorporeal membrane lung (ECML) would provide means to manipulate the metabolic rate in sheep.

**Methods:**

A partial venoarterial cardiopulmonary bypass was established in anesthetized, ventilated (fraction of inspired oxygen = 0.5) sheep. The ECML was alternately ventilated with air or air containing 100, 200, or 300 ppm H_2_S for intervals of 1 hour. Metabolic rate was estimated on the basis of total CO_2 _production (${\dot{\text{V}}\text{CO}}_{\text{2}}$) and O_2 _consumption (${\dot{\text{V}}\text{O}}_{\text{2}}$). Continuous hemodynamic monitoring was performed via indwelling femoral and pulmonary artery catheters.

**Results:**

${\dot{\text{V}}\text{CO}}_{\text{2}}$, ${\dot{\text{V}}\text{O}}_{\text{2}}$, and cardiac output ranged within normal physiological limits when the ECML was ventilated with air and did not change after administration of up to 300 ppm H_2_S. Administration of 100, 200 and 300 ppm H_2_S increased pulmonary vascular resistance by 46, 52 and 141 dyn·s/cm^5^, respectively (all *P *≤ 0.05 for air vs. 100, 200 and 300 ppm H_2_S, respectively), and mean pulmonary artery pressure by 4 mmHg (*P *≤ 0.05), 3 mmHg (n.s.) and 11 mmHg (*P *≤ 0.05), respectively, without changing pulmonary capillary wedge pressure or cardiac output. Exposure to 300 ppm H_2_S decreased systemic vascular resistance from 1,561 ± 553 to 870 ± 138 dyn·s/cm^5 ^(*P *≤ 0.05) and mean arterial pressure from 121 ± 15 mmHg to 66 ± 11 mmHg (*P *≤ 0.05). In addition, exposure to 300 ppm H_2_S impaired arterial oxygenation (P_a_O_2 _114 ± 36 mmHg with air vs. 83 ± 23 mmHg with H_2_S; *P *≤ 0.05).

**Conclusions:**

Administration of up to 300 ppm H_2_S via ventilation of an extracorporeal membrane lung does not reduce ${\dot{\text{V}}\text{CO}}_{\text{2}}$ and ${\dot{\text{V}}\text{O}}_{\text{2}}$, but causes dose-dependent pulmonary vasoconstriction and systemic vasodilation. These results suggest that administration of high concentrations of H_2_S in venoarterial cardiopulmonary bypass circulation does not reduce metabolism in anesthetized sheep but confers systemic and pulmonary vasomotor effects.

## Introduction

Balancing cellular oxygen supply and demand is a key therapeutic approach to protecting organs such as the brain, kidneys and heart from ischemic injury. Permissive hypothermia and active cooling have been shown to reduce oxygen demands in patients experiencing stroke, cardiac arrest, cardiac surgery, severe trauma and other instances of ischemia and subsequent reperfusion [1-4]. However, hypothermic reduction of aerobic metabolism has been associated with adverse effects, including increased rates of infection and coagulopathy [5,6]. Developing other methods to acutely reduce metabolism in patients could be clinically useful.

Hydrogen sulfide (H_2_S) is an inhibitor of cytochrome C oxidase in the mitochondrial electron transport chain [7] that reduces metabolism and body temperature in mice and rats [8,9]. Inhalation of H_2_S or intravenous administration of H_2_S donor compounds (NaHS or Na_2_S) can protect rodents from hypoxia [10] or hemorrhagic shock [11], improve survival rates after cardiac arrest and cardiopulmonary resuscitation in mice [12], and attenuate myocardial ischemia-reperfusion injury in both rodents [13] and pigs [14].

Although inhaling H_2_S at 60 to 80 ppm reduces metabolism in mice, it has been reported that inhaled H_2_S does not depress total CO_2 _production (${\dot{\text{V}}\text{CO}}_{\text{2}}$) and total O_2 _consumption (${\dot{\text{V}}\text{O}}_{\text{2}}$) in sedated, spontaneously breathing sheep (60 ppm H_2_S) [15] or anesthetized, ventilated piglets (20 to 80 ppm H_2_S) [16]. On the other hand, Struve *et al*. [8] reported that inhalation of H_2_S at 200 to 400 ppm, but not at 30 to 80 ppm, decreased body temperature in rats. Similarly, Morrison *et al*. [11] showed that inhaling H_2_S at 300 ppm was required to decrease ${\dot{\text{V}}\text{CO}}_{\text{2}}$ in rats, in contrast to 80 ppm in mice. While these observations suggest that higher levels of H_2_S are likely to be required to alter metabolic rates in larger animals [11], the effects of higher concentrations of H_2_S on metabolism in larger mammals have not been examined.

It is well documented, however, that inhalation of high concentrations of H_2_S may injure the bronchial mucosa, cause pulmonary edema, and impair gas exchange [17,18]. To examine the impact of delivering higher concentrations of H_2_S to the body without incurring the pulmonary toxicity of H_2_S inhalation, we administered H_2_S gas via an extracorporeal membrane lung (ECML). We hypothesized that high concentrations of H_2_S delivered via ECML in a partial venoarterial bypass system delivering blood to the aortic root might reduce the metabolic rate in sheep at rest. If ECML ventilation with H_2_S was found to reduce the metabolic rate in sheep, this method might provide a novel approach to balance the supply and demand of oxygen in a variety of situations, including in those patients who are supported by extracorporeal circulation during cardiac surgery or severe acute respiratory distress.

## Materials and methods

All procedures described here were approved by the Subcommittee on Research Animal Care of the Massachusetts General Hospital, Boston, MA, USA, and adhered to the principles of the Declaration of Helsinki and the Recommendations for the Care and Use of Animals.

### Animal housing and maintenance

Five female purebred Polypay sheep (body weight: 30.6 ± 2.5 kg, mean ± SD) were obtained from a single-source breeder (New England Ovis LLC, Rollinsford, NH, USA) and were housed under standard environmental conditions (air-conditioned room at 22°C, 50% relative humidity, 12-hour light-dark cycle) for at least 5 days prior to each study. Animals were fed standard chow (Rumilab diet 5508; PMI Feeds Inc., St. Louis, MO, USA) twice daily and were fasted for 24 hours with free access to water before each experiment.

### Instrumentation

After intramuscular premedication with 5 mg/kg ketamine (ketamine hydrochloride; Hospira Inc., Lake Forest, IL, USA) and 0.1 mg/kg xylazine (Anased; Lloyd Laboratories, Shenandoah, IA, USA), a venous cannula (Surflo IV catheter 18G; Terumo, Elkton, MD, USA) was inserted into an ear vein and a bolus of 0.1 to 0.2 mg/kg diazepam (Diazepam USP; Hospira, Lake Forest, IL, USA) administered intravenously (iv). Subsequently, the animals were placed in a supine position and were intubated and mechanically ventilated with a volume-controlled mode (fraction of inspired oxygen (F_i_O_2_) 50%, tidal volume 10 ml/kg) (7200 Series Ventilator System; Puritan Bennett, Boulder, CO, USA). Anesthesia was maintained by a constant rate infusion of ketamine at 3 mg∙kg^-1^∙h^-1 ^and diazepam at 0.5 mg∙kg^-1^∙h^-1^. Respiratory rate was adjusted to maintain the end-tidal CO_2 _between 35 and 40 mmHg. An arterial catheter (18G, FA-04018; Arrow Inc., Reading, PA, USA) was placed into the right femoral artery via percutaneous puncture to monitor mean arterial pressure (MAP) and to sample blood. Subsequently, an 8-Fr heptalumen pulmonary artery catheter (746HF8; Edwards Lifesciences, Irvine, CA, USA) was introduced through a percutaneous sheath (9 Fr, PB-09903; Arrow Inc., Reading, PA, USA) into the left external jugular vein for blood sampling and monitoring of mean pulmonary artery pressure (MPAP), central venous pressure (CVP), pulmonary capillary wedge pressure (PCWP), continuous cardiac output (CO) and blood temperature. Finally, a transurethral bladder catheter and a transesophageal gastric tube were inserted to drain urine and gastric secretions. During the first hour after induction, animals received an infusion of 500 ml of 6% hetastarch (Hextend; Hospira, Lake Forest, IL, USA) and 500 ml of lactated Ringer's solution (Baxter, Deerfield, IL, USA); thereafter, 16 ml∙kg^-1^∙h^-1 ^of lactated Ringer's solution and 9 ml∙kg^-1^∙h^-1 ^of 0.9% saline were infused to match fluid losses from diuresis and gastric secretions.

### Extracorporeal circulation

A 20-Fr single-stage venous cannula (DLP; Medtronic, Minneapolis, MN, USA) and a 14-Fr arterial cannula (Fem-Flex II; Medtronic) were surgically inserted and advanced through the right external jugular vein and right common carotid artery, respectively, thereby enabling blood withdrawal from the superior vena cava and arterial blood return to the aortic root from the extracorporeal cardiopulmonary bypass circuit. The bypass circuit comprised a three-eighths-inch polyethylene tubing line (3506; Medtronic), an occlusive roller pump (Cardiovascular Instruments Corp., Wakefield, MA, USA) and an ECML (Trillium 541TT Affinity; Medtronic) with an integral heat exchanger, and it was primed with a total extracorporeal priming volume of 500 ml of 0.9% saline. A bolus injection of unfractionated heparin (200 IU/kg heparin sodium; APP Pharmaceuticals, LLC, Schaumburg, IL, USA) prior to cannulation, followed by a continuous infusion of 200 IU/kg unfractionated heparin per hour was used for anticoagulation. A thermostat-controlled water bath (Haake DC10-P5; Thermo Scientific, Waltham, MA, USA) supplying the heat exchanger with circulating water was maintained at 38°C. The gas compartment of the oxygenator was ventilated at a constant flow of 5 l/min with oxygen, air and H_2_S (10,000 ppm hydrogen sulfide balanced with nitrogen; Airgas Specialty Gases, Port Allen, LA, USA) blended to achieve an oxygen concentration of 21% with 0, 100, 200, or 300 ppm H_2_S.

A handheld iTX Multi-Gas detector (1 ppm detection threshold; Industrial Scientific, Oakdale, PA, USA) was used to monitor the H_2_S concentrations at the inlet and outlet of the gas compartment.

### Experimental procedures

Once partial venoarterial bypass perfusion was started, the transmembrane blood flow was gradually increased to 1 l/min. Then the respiratory rate was reduced to maintain an end-tidal partial pressure of CO_2 _of 35 to 40 mmHg, and sheep were paralyzed (0.1 mg∙kg^-1^∙h^-1 ^of pancuronium bromide iv; Sicor Pharmaceuticals, Irvine, CA, USA) to prevent spontaneous respiratory activity, asynchronous ventilation and excessive skeletal muscle O_2 _consumption. A 1-hour equilibration period was allowed to achieve hemodynamic stability before baseline measurements were taken.

During the following 6 hours, the ECML gas compartment was alternately ventilated with either air or air plus H_2_S for 1-hour intervals, thereby administering 0 ppm H_2_S during the first hour, 100 ppm H_2_S during the second hour, followed by 0 and 200 ppm during the third and fourth hours and finally 0 and 300 ppm H_2_S during the fifth and sixth hours. This procedure was chosen to detect the hemodynamic and metabolic effects of exposure to increasing H_2_S concentrations through the membrane lung, as well as their reversibility.

### Measurements and monitoring

A digital data acquisition system (PowerLab and Chart software version 5.0; ADInstruments, Colorado Springs, CO, USA) was used to continuously record MAP, CVP and MPAP. A Vigilance II Monitor (Edwards Lifesciences) was used to continuously measure CO and central blood temperature. End-tidal CO_2_, as well as the total amount of CO_2 _exhaled from the biological lungs per unit of time (${\dot{\text{V}}}_{\text{L}}{\text{CO}}_{\text{2}}$), was measured by an in-stream, noninvasive, continuous monitoring device (NICO Cardiopulmonary Management System; Philips Respironics, Murrysville, PA, USA). Blood gas tensions, hemoglobin concentrations, and acid-base balances were determined in arterial and mixed venous blood samples using a standard blood gas analyzer (ABL 800 Flex; Radiometer, Copenhagen, Denmark).

Plasma concentrations of H_2_S were measured in duplicate as total sulfide concentrations using the methylene blue formation method with modifications [19]. Briefly, arterial and ECML-efferent blood was sampled and immediately centrifuged at 4°C to obtain plasma. An aliquot of plasma (100 μl) was added with 2% zinc acetate (200 μl) to trap the H_2_S, and 10% trichloroacetic acid (200 μl) was added to precipitate plasma proteins, immediately followed by 20 mM *N*,*N*-dimethyl-1,4-phenylenediamine sulfate in 7.2 M HCl (100 μl) and 30 mM FeCl_3 _in 1.2 M HCl (100 μl). The reaction mixture was incubated for 20 minutes at room temperature and centrifuged at 14,000 rpm for 10 minutes. The absorbance of the supernatant was measured at 670 nm using a spectrophotometer. Total sulfide concentration was calculated against a standard curve made with known concentrations of Na_2_S solutions in phosphate-buffered saline. The lower detection limit of this assay was approximately 1 μM sulfide in plasma.

### Calculation of carbon dioxide production

Total ${\dot{\text{V}}\text{CO}}_{\text{2}}$ was monitored continuously and was calculated as the sum of CO_2 _exhaled from the lungs per unit of time (${\dot{\text{V}}}_{\text{L}}{\text{CO}}_{\text{2}}$) and the amount of CO_2 _removed from the circulation via the membrane oxygenator (${\dot{\text{V}}}_{\text{M}}{\text{CO}}_{\text{2}}$), according to the following equations:

(1) $${\dot{\text{V}}}_{\text{L}}{\text{CO}}_{\text{2}}={\dot{\text{V}}}_{\text{E}}\times F_{\text{E}}{\text{CO}}_{\text{2}},$$

where ${\dot{\text{V}}}_{\text{E}}$ is the expiratory minute volume and *F*_E_CO_2 _is the mean fraction of CO_2 _in expired air. Quantification of ${\dot{\text{V}}}_{\text{E}}$ and *F*_E_CO_2 _and the calculation of ${\dot{\text{V}}}_{\text{L}}{\text{CO}}_{\text{2}}$ were accomplished by a continuous noninvasive NICO device (see 'Measurements and monitoring' section):

(2) $${\dot{\text{V}}}_{\text{M}}{\text{CO}}_{\text{2}}={\text{Q}}_{\text{gas}}\times F_{\text{M}}{\text{CO}}_{\text{2}},$$

where Q_gas _is the total gas flow exhausted from the membrane oxygenator and *F*_E_CO_2 _is the fraction of CO_2 _in the exhaust gas. Q_gas _was continuously monitored by a microturbine flow meter (S-113 Flo-Meter; McMillan Co., Georgetown, TX, USA), and *F*_E_CO_2 _was measured by a sidestream infrared CO_2 _analyzer (WMA-4; PP-Systems, Amesbury, MA, USA).

### Calculation of oxygen consumption

Total ${\dot{\text{V}}\text{O}}_{\text{2}}$ was calculated on the basis of blood samples drawn 10 minutes before the end of each period of exposure to air or H_2_S as follows:

(3) $${\dot{\text{V}}\text{O}}_{\text{2}}={\text{(c}}_{\text{a}}{\text{O}}_{\text{2}}{\text{-c}}_{\text{v}}{\text{O}}_{\text{2}}\text{)}\times {\text{Q}}_{\text{L}}{\text{-(c}}_{\text{e}}{\text{O}}_{\text{2}}{\text{-c}}_{\text{a}}{\text{O}}_{\text{2}}\text{)}\times {\text{Q}}_{\text{M}},$$

where c_a_O_2 _is the oxygen content of arterial blood, c_v_O_2 _is the oxygen content of mixed venous blood, Q_L _is transpulmonary blood flow (here meaning continuous CO measured via pulmonary artery catheter), c_e_O_2 _is the oxygen content of ECML-efferent blood and Q_M _is extrapulmonary blood flow (here meaning transmembrane blood flow). Blood oxygen content (cO_2_) was calculated according to the following general equation:

(4) $${\text{cO}}_{\text{2}}=\text{[Hb]}\times F{\text{O}}_{\text{2}}\text{Hb}\times \text{1}\text{.34}+{\text{pO}}_{\text{2}}\times \text{0}\text{.003},$$

where [Hb] is the hemoglobin concentration, *F*O_2_Hb is the fraction of oxyhemoglobin, 1.34 is Hüfner's constant and pO_2 _is the oxygen tension.

### Statistical analysis

**Figure 1:**
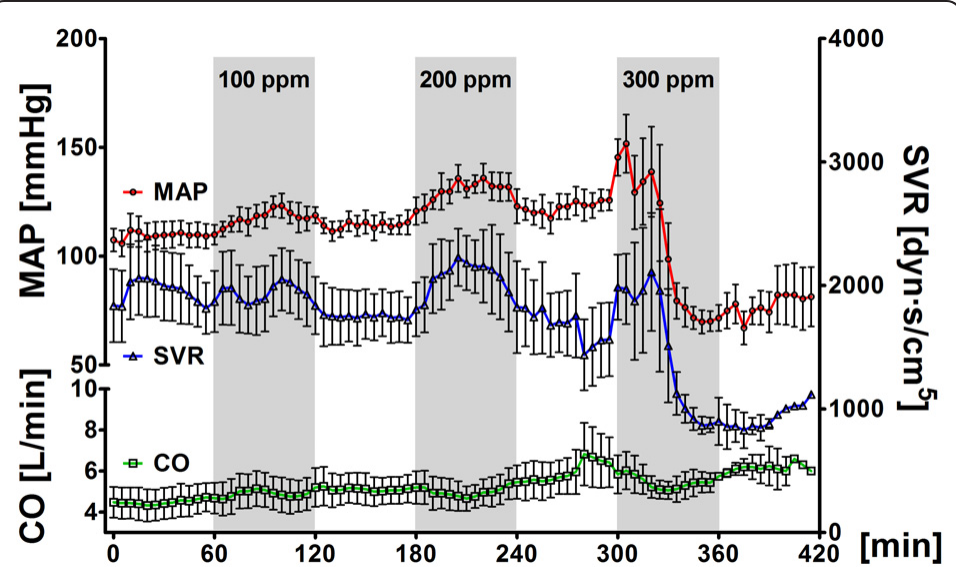
**Systemic vascular hemodynamics**. Systemic vascular hemodynamics in five sheep challenged with alternate exposure to hydrogen sulfide (H_2_S) (gray bars) by ventilation of an extracorporeal membrane lung with 0 or 100 ppm H_2_S in air, 200 ppm H_2_S in air and 300 ppm H_2_S in air for 1-hour intervals each. Data are presented as means ± standard error of the mean. MAP, mean arterial pressure; CO, cardiac output; SVR, systemic vascular resistance; ppm, parts per million.

**Figure 2:**
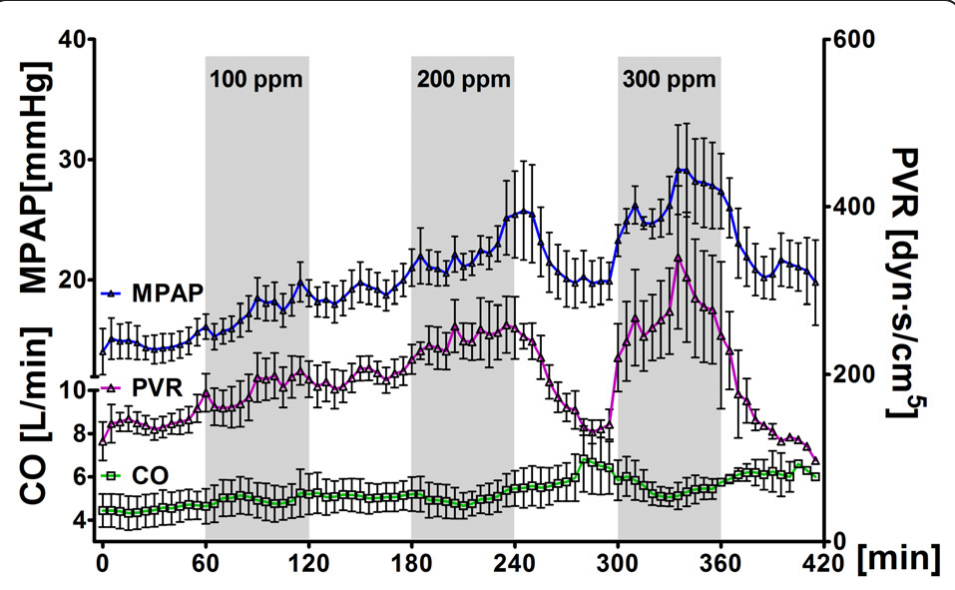
**Pulmonary vascular hemodynamics**. Pulmonary vascular hemodynamics in five sheep challenged with alternate exposure to hydrogen sulfide (H_2_S) (gray bars) by ventilation of an extracorporeal membrane lung with 0 or 100 ppm H_2_S in air, 200 ppm H_2_S in air and 300 ppm H_2_S in air for 1-hour intervals each. Data are presented as means ± standard error of the mean. MPAP, mean pulmonary artery pressure; CO, cardiac output; PVR, pulmonary vascular resistance; ppm, parts per million.

Statistical analysis was performed using the SPSS 14.0 data package for Windows (SPSS, Chicago, IL, USA) and GraphPad Prism version 5.02 software (GraphPad Software, La Jolla, CA, USA). All data are reported as means ± SD unless indicated otherwise. Hemodynamic parameters, ${\dot{\text{V}}\text{CO}}_{\text{2}}$ and body temperature were measured continuously and are reported as the mean value derived from the last 10 minutes of each period of exposure to air or H_2_S. In addition, hemodynamic parameters were averaged every 5 minutes for a time course analysis, and these data are displayed in Figures 1 and 2. Blood gas tension analysis, determination of blood hemoglobin concentrations and quantification of H_2_S plasma concentrations required blood sampling. Samples were obtained during the last 5 minutes of each period of exposure. Depending on the distribution of the data as determined using the Shapiro-Wilk test for normal distribution, either Student's *t*-test or the Wilcoxon signed-rank test was performed to compare each H_2_S ventilation period with the respective baseline period (0 ppm H_2_S). Statistical significance was assumed at *P *≤ 0.05. On the basis of data derived from pilot experiments, power and sample size calculations were performed using PS: Power and Sample Size Calculation version 2.1.31 software by Dupont and Plummer [20].

## Results

### Metabolic effects of H_2_S administration

**Figure 3:**
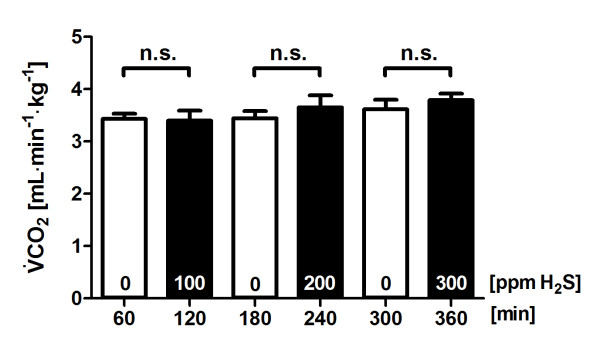
**Carbon dioxide production during administration of hydrogen sulfide (H**_**2**_**S)**. Total carbon dioxide production (${\dot{\text{V}}\text{CO}}_{\text{2}}$) in five sheep challenged with alternate exposure to H_2_S by ventilation of an extracorporeal membrane lung with 0 or 100 ppm H_2_S in air, 200 ppm H_2_S in air and 300 ppm H_2_S in air for 1-hour intervals each. Values are derived from the last 10 minutes of each period of exposure to air or H_2_S and are presented as means ± standard error of the mean. ppm, parts per million; n.s. = *P *> 0.05.

**Figure 4:**
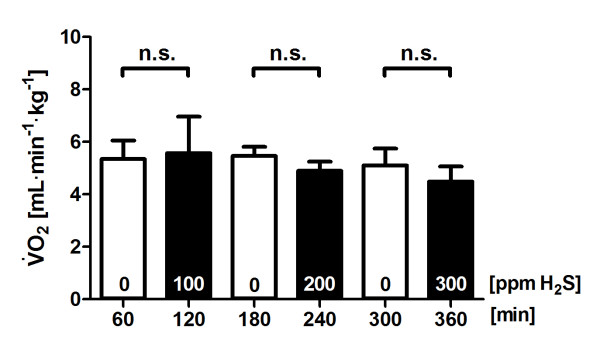
**Oxygen consumption during administration of hydrogen sulfide (H**_**2**_**S)**. Total carbon dioxide production (${\dot{\text{V}}\text{O}}_{\text{2}}$) in five sheep challenged with alternate exposure to H_2_S by ventilation of an extracorporeal membrane lung with 0 or 100 ppm H_2_S in air, 200 ppm H_2_S in air and 300 ppm H_2_S in air for 1-hour intervals each. Values are derived from blood samples taken during the last 10 minutes of each period of exposure to air or H_2_S and are presented as means ± standard error of the mean. ppm, parts per million; n.s. = *P *> 0.05.

The baseline ${\dot{\text{V}}\text{CO}}_{\text{2}}$ value was stably near approximately 3.4 ml∙kg^-1^∙min^-1 ^when the ECML was ventilated with air. Direct diffusion of H_2_S into blood via the ECML at 100, 200 or 300 ppm did not alter ${\dot{\text{V}}\text{CO}}_{\text{2}}$ (Figure 3) or ${\dot{\text{V}}\text{O}}_{\text{2}}$(Figure 4). The temperature of the ECML heat exchanger water bath was kept at 38°C and resulted in a constant central blood temperature of 37.4 ± 0.4°C throughout the experiment (Table 1).

**Table 1:** Hemodynamics and blood gas data^a^

Parameter	0 ppm	100 ppm	0 ppm	200 ppm	0 ppm	300 ppm
Hemodynamics, means ± SD						
HR, beats/min	139 ± 24	148 ± 29	154 ± 5	172 ± 28	165 ± 28	150 ± 31
MAP, mmHg	110 ± 13	117 ± 14	115 ± 11	128 ± 16	121 ± 15	66 ± 11^b^
MPAP, mmHg	15 ± 3	19 ± 3*	19 ± 3	22 ± 4	20 ± 4.0	31 ± 7^b^
CO, l/min	4.6 ± 1.4	4.9 ± 2.0	5.1 ± 1.5	5.2 ± 1.7	5.8 ± 2.3	5.5 ± 1.2
CVP, mmHg	9 ± 2	9 ± 1.0	10 ± 1	11 ± 2	11 ± 1	11 ± 2
PCWP, mmHg	7 ± 2	7 ± 2	7 ± 8	8 ± 2	9 ± 2	10 ± 2
SVR, dyn·s/cm^5^	1,843 ± 435	1,948 ± 525	1,734 ± 412	2,009 ± 703^b^	1,561 ± 553	870 ± 138^b^
PVR, dyn·s/cm^5^	145 ± 32	191 ± 52^b^	203 ± 36	255 ± 70^b^	138 ± 27	279 ± 138^b^
Hb, pH, blood gas tensions, and temperature, means ± SD						
Hb_a_, g/dl	8.6 ± 1.3	9.0 ± 1.3	9.1 ± 1.0	11.1 ± 1.4^b^	9.5 ± 0.6	9.6 ± 1.2
pH_a_	7.401 ± 0.072	7.369 ± 0.079	7.375 ± 0.051	7.346 ± 0.063	7.312 ± 0.089	7.217 ± 0.064^b^
P_a_O_2_, mmHg	161 ± 28	150 ± 40	150 ± 37	107 ± 39	114 ± 36	83 ± 23^b^
P_a_CO_2_, mmHg	38 ± 13	38 ± 11	35 ± 7	34 ± 5	36 ± 7.0	38 ± 4
pH_v_	7.383 ± 0.074	7.360 ± 0.080	7.360 ± 0.056	7.346 ± 0.066	7.302 ± 0.087	7.210 ± 0.068^b^
P_v_O_2_, mmHg	50 ± 5	52 ± 6^b^	52 ± 4	54 ± 4	56 ± 4	52 ± 7
P_v_CO_2_, mmHg	41 ± 14	41 ± 11	38 ± 8	35 ± 5	38 ± 6	40 ± 4
Temperature,°C	37.5 ± 0.6	37.5 ± 0.4	37.5 ± 0.3	37.3 ± 0.4	37.3 ± 0.4	37.1 ± 0.5

^a^Hemodynamics and blood gas data in five sheep challenged with alternate exposure to H_2_S by ventilation of an extracorporeal membrane lung with 0 or 100 ppm H_2_S, 200 ppm H_2_S or 300 ppm H_2_S in air for 1-hour intervals each. ppm, parts per million; HR, heart rate; MAP, mean arterial pressure; MPAP, mean pulmonary artery pressure; CO, cardiac output; CVP, central venous pressure; PCWP, pulmonary capillary wedge pressure; SVR, systemic vascular resistance; PVR, pulmonary vascular resistance; Hb_a_, arterial hemoglobin concentration; pH_a_, arterial pH; P_a_O_2_, arterial oxygen tension; P_a_CO_2_, arterial carbon dioxide tension; pH_v_, mixed venous pH; P_v_O_2_, mixed venous oxygen tension; P_v_CO_2_, mixed venous carbon dioxide tension. All values are means ± SD and reflect the last 10 minutes of each 1-hour period. *n *= 5. Values during H_2_S exposure were compared using Student's *t*-test or the Wilcoxon signed-rank test with the preceding 0 ppm baseline period, that is, first vs. second hour, third vs. fourth hour and fifth vs. sixth hour; ^b^*P *≤ 0.05.

### Hemodynamic effects of H_2_S administration

After 1 hour of exposure to either 100 or 200 ppm H_2_S via ECML ventilation and partial venoarterial perfusion, MAP was not different from baseline. However, exposure to 300 ppm H_2_S for 1 hour decreased MAP from 121 ± 15 mmHg to 66 ± 11 mmHg and reduced systemic vascular resistance (SVR) from 1561 ± 553 dyn·s/cm^5 ^to 870 ± 138 dyn·s/cm^5 ^(Table 1). We noted that MAP increased transiently during exposure to 100 and 200 ppm H_2_S (Figure 1) and that this increase was rapidly reversed upon application of air without added H_2_S. Subsequently, exposure to 300 ppm H_2_S induced a biphasic systemic pressor response characterized by increased MAP and SVR during the first 20 minutes of H_2_S exposure followed by a rapid decrease of MAP and pronounced irreversible hypotension (Figure 1).

MPAP and pulmonary vascular resistance (PVR) increased in response to H_2_S exposure, with the greatest increase (ΔMPAP, approximately 10 mmHg; ΔPVR, +51%) observed in response to 300 ppm H_2_S (Table 1). Time course analysis (Figure 2) suggested that PVR increased after exposure to 100, 200 and 300 ppm H_2_S in a reversible, dose-dependent manner. Heart rate and CO did not change in response to H_2_S exposure.

### Pulmonary gas exchange and acid-base status

Arterial CO_2 _tension levels were within physiological limits throughout the experiment and did not change in response to H_2_S. Mixed venous CO_2 _tension (P_v_CO_2_) ranged between 35 and 41 mmHg and did not change in response to H_2_S. While arterial oxygenation (P_a_O_2_) was not significantly affected by 100 or 200 ppm H_2_S, P_a_O_2 _decreased from 114 ± 36 to 83 ± 23 mmHg (*P *≤ 0.05) upon administration of 300 ppm H_2_S. Arterial oxygen tension did not recover during the subsequent interval of air exposure without H_2_S. Mixed venous O_2 _tension ranged between 50 and 56 mmHg, and there was no relevant change upon H_2_S administration. While arterial pH (pH_a_) was within physiological limits throughout the experiment, significant metabolic acidosis was observed during exposure to 300 ppm H_2_S, with concomitant changes in mixed venous pH. Arterial hemoglobin concentrations were near 9 g/dl throughout the experiment. Exposure to 200 ppm H_2_S transiently increased hemoglobin concentrations by 2 ± 0 g/dl (Table 1).

### Total plasma sulfide concentrations

Plasma sulfide concentrations were determined in duplicate from arterial and ECML-efferent blood. The baseline plasma concentration of sulfide was 1.9 ± 0.3 μM, and this value was only slightly higher than the lower detection limit (approximately 1 μM) for this assay. Ventilation of ECML with air did not affect plasma sulfide concentrations in the efferent blood of the ECML. In ECML-efferent blood, plasma sulfide concentration increased to 7 ± 6, 27 ± 6 and 62 ± 12 μM/l during ECML ventilation with 100, 200 and 300 ppm H_2_S, respectively. However, no sulfide was detected in plasma samples of blood collected from the femoral artery during exposure to 100, 200 or 300 ppm H_2_S.

## Discussion

The results of the present study reveal that ventilating an ECML with up to 300 ppm H_2_S in venoarterial cardiac bypass circulation does not reduce whole body CO_2 _production or O_2 _consumption in anesthetized sheep. In addition, we have demonstrated that administration of 300 ppm H_2_S via ECML ventilation causes significant adverse effects, including pulmonary vasoconstriction, systemic vasodilation and hypoxemia. The current results do not support the hypothesis that high concentrations of H_2_S delivered via an ECML can reduce the metabolic rate in large mammals at rest.

In an attempt to bypass the direct pulmonary toxicity of inhaled H_2_S, we used an ECML to directly diffuse high concentrations of H_2_S gas into the blood. The absence of H_2_S (lower limit of detection 1 ppm) in the gas outlet of the artificial lung during ventilation with up to 300 ppm H_2_S indicates that H_2_S is highly diffusible into blood through the membrane and that a single passage is sufficient for complete uptake of the gas. Thus, assuming complete uptake of H_2_S during ventilation of the ECML at a gas flow of 5 l/min with 300 ppm H_2_S (at standard conditions for temperature and pressure), a total amount of 1.5 ml of H_2_S (that is, approximately 67 μM) are administered via the membrane every minute. This sums to about 134 μM H_2_S/kg per hour delivered to a 30-kg sheep in the current study. In contrast, the total amount of H_2_S administered in previous studies in sheep [15] and pigs [16] were approximately 13 μM/kg/h and approximately 42 μM/kg/h, respectively, assuming complete uptake of H_2_S from the alveolar space and an alveolar ventilation of 6 l/min in a 74-kg sheep, and 1.2 l/min in a 6-kg pig. Therefore, the systemic dose of H_2_S supplied in the present study was about three times greater than that applied in pigs and 10 times greater than the dose applied in sheep. If any of the alveolar H_2_S were exhaled, the ratio of the uptake via the membrane artificial lung in the present study and the uptake via the natural lungs in previous reports would be even greater. Nonetheless, our measurements suggest that administration of H_2_S up to 134 μM/kg/h does not reduce ${\dot{\text{V}}\text{CO}}_{\text{2}}$ or ${\dot{\text{V}}\text{O}}_{\text{2}}$ in sheep.

While H_2_S did not reduce ${\dot{\text{V}}\text{CO}}_{\text{2}}$ or ${\dot{\text{V}}\text{O}}_{\text{2}}$ in sheep in the present study, Simon *et al*. [21] reported that continuous iv infusion of Na_2_S for 8 hours decreased the core body temperature and ${\dot{\text{V}}\text{CO}}_{\text{2}}$ and ${\dot{\text{V}}\text{O}}_{\text{2}}$ levels in pigs, suggesting that it is possible to reduce metabolic rates in large mammals using a sulfide-based approach. However, it is important to note that hypothermia itself reduces the metabolic rate (*Q*_10 _effect). Therefore, in the current study, body temperature was kept at 37°C throughout the experiment to exclude any effects of hypothermia on metabolism. Whether systemic administration of Na_2_S reduces metabolic rates in large mammals when normothermia is maintained remains to be determined.

While our findings support the inability of H_2_S to reduce metabolism in large mammals, these results differ from observations in mice in which H_2_S inhalation markedly reduced metabolism [9,10,22]. Hydrogen sulfide may be one, but not the only, trigger for murine metabolic depression. Indeed, hypoxia, anemia and exposure to carbon monoxide have been reported to reduce aerobic metabolism in mice [23-25], but not in large mammals [26-28]. Of note is that mice are known to have a much higher specific metabolic rate (approximately 168 kcal kg^-1^∙d^-1 ^in a 30-g mouse) than sheep (approximately 30 kcal kg^-1^∙d^-1 ^in a 30-kg sheep) [29]. In a previous study, we reported that H_2_S inhalation reduced metabolism in awake, spontaneously breathing mice by about 40% during normothermia, resulting in a specific metabolic rate of no more than approximately 100 kcal∙kg^-1^∙d^-1 ^[9]. In contrast, it has been reported that H_2_S inhalation at 100 ppm failed to reduce CO_2 _production in normothermic mice that were anesthetized and mechanically ventilated [30]. Interestingly, in anesthetized mice studied by Baumgart *et al*. [30], the baseline CO_2 _production rate before H_2_S inhalation was approximately 50% less than that in awake mice studied by Volpato *et al*. [9] in our laboratory. It is tempting to speculate that the ability of H_2_S to reduce metabolism depends on the specific metabolic rate of animals. H_2_S may reduce metabolism when the baseline rate of metabolism is high (for example, in awake mice), but not when the metabolic rate is already depressed (for example, in anesthetized mice or sheep).

Along these lines, it may be possible to reduce the metabolic rate in larger mammals using H_2_S when metabolism is increased. It has been reported that inhalation of 10 ppm H_2_S reduced oxygen consumption in exercising healthy volunteers, presumably due to inhibition of aerobiosis in exercising muscle [31]. Inhibitory effects of H_2_S in the presence of increased metabolism in larger mammals warrants further study.

Our results show that administration of H_2_S via a cardiopulmonary bypass circulation can cause significant dose-dependent pulmonary vasoconstriction. These observations are consistent with the pulmonary vasoconstrictor effects of H_2_S in mammalian pulmonary vessels reported by Olson *et al*. [32]. Although a potential role of H_2_S in hypoxia sensing (hence hypoxic pulmonary vasoconstriction) has been suggested [33], the mechanisms responsible for the pulmonary vasoconstrictor effects of H_2_S remain to be further elucidated.

Administration of H_2_S also tended to increase systemic vascular resistance, but resulted in systemic vasodilation after 30 minutes of ECML ventilation with 300 ppm H_2_S. This is consistent with previous reports demonstrating that H_2_S can produce both vasoconstriction and vasorelaxation in isolated rat aortic ring segments in an O_2 _concentration-dependent manner. Koenitzer *et al*. [34] reported that H_2_S (5 to 80 μM Na_2_S solution) causes vasorelaxation at O_2 _concentrations reflecting the physiological oxygen tension in the peripheral vasculature (O_2 _concentration, 40 μM). In contrast, at high O_2 _concentrations (O_2_, 200 μM) under which H_2_S is rapidly oxidized to sulfite, sulfate or thiosulfate, the administration of 5 to 100 μM Na_2_S causes rat aortic vasoconstriction, and more than 200 μM Na_2_S are required to cause vasorelaxation [34]. Along these lines, the high oxygen tension observed in sheep on ECML when ventilated with 100 and 200 ppm of H_2_S may have contributed to the systemic vasoconstrictor effects of H_2_S in the present study, whereas vasodilation was only observed at the highest H_2_S concentration (300 ppm). In addition, the O_2 _dependency of H_2_S-mediated vasoconstriction may also explain why H_2_S caused vasoconstriction in the pulmonary vasculature, where O_2 _availability is consistently high.

While the toxicity of inhaling high levels of H_2_S is well documented, the reported toxicity of H_2_S concentrations up to 500 ppm is almost exclusively limited to mucosal membranes and the central nervous system [35-37]. However, the cardiovascular toxicity of high levels of inhaled H_2_S has not been reported. The observed pulmonary hypertension and apparent changes in systemic vascular tone in the current study may therefore represent previously unrecognized toxic effects of high levels of H_2_S in the circulation.

Despite the availability of various methods used to quantify sulfide in biological fluids, it remains challenging to measure circulating plasma concentrations of H_2_S [38]. The methylene blue formation method employed here measures "labile" total sulfide liberated from sulfur compounds, but not free H_2_S in blood and tissue. In the current study, considerable sulfide concentrations were detected in plasma obtained from blood efferent from the ECML, but not in the blood samples from the femoral artery (sampled less than approximately 10 seconds after the blood left the ECML). These observations suggest a rapid uptake of H_2_S into a variety of sulfide pools once H_2_S has entered the blood stream. Of note is that the measured plasma sulfide level of 62 μM/l in the ECML efferent blood diffused with 300 ppm H_2_S was only about 3% of the expected sulfide level of approximately 2,000 μM/l assuming a blood volume of 70 ml/kg. These results are consistent with a recent report that circulating free sulfide levels are almost undetectably low at baseline and that exogenous sulfide is also rapidly removed from the circulating plasma [39]. Nonetheless, the pronounced vasoreactivity induced by H_2_S administration observed in the current study suggests that H_2_S (and/or its active metabolites) is transported to the periphery and exerts biological effects. The fate of exogenously administered H_2_S remains to be determined in future studies using more sensitive methods.

Although the results of the current study do not suggest that H_2_S can be used to reduce metabolic rate in larger mammals, these results do not refute the potential organ protective effects of H_2_S reported elsewhere. The dose of 134 μM/kg/h that was applied here is almost 20 times higher than the effective dose of Na_2_S reported to improve survival in mice after cardiac arrest (0.55 μg/g, that is, approximately 7 μM/kg) [12]. Studies by others have also shown that administration of H_2_S donors in a similarly low dose range were able to protect organs from ischemic insults in rodents and pigs without reducing metabolic rate or body temperature [14,40]. Taken together, it is conceivable that organ-protective effects and metabolic effects of H_2_S may be mediated via two different mechanisms and/or at different concentrations.

### Limitations

Measuring oxygen consumption is a valuable tool to assess metabolic rate. However, quantification of oxygen consumption in the setting of ECML requires serial simultaneous determinations of oxygen content in arterial and mixed venous blood as well as blood afferent and efferent to the ECML [41]. Small measuring inaccuracies in the parameters needed to calculate oxygen content (hemoglobin, oxygen saturation and tension) result in an exponential increase in the overall inaccuracy of the calculated ${\dot{\text{V}}\text{O}}_{\text{2}}$ value. In contrast, measuring CO_2 _production requires only CO_2 _quantification in the exhaled gas of both the natural and the artificial lung because virtually no CO_2 _is present in the inhaled gas mixture, which is a major advantage to simplifying the setup and avoiding exponential error. Therefore, ${\dot{\text{V}}\text{CO}}_{\text{2}}$ may be the more reliable index for estimating the metabolic rate in this study.

The present study was designed to detect a reduction in metabolic rate of about 30% in sheep. On the basis of the variance of metabolic rates determined in pilot experiments in sheep, a sample size of 12 sheep was calculated to find a 30% reduction in metabolic rate (80% power and 5% probability of error). An interim analysis of this study (*n *= 5) did not substantiate a significant change or trend in ${\dot{\text{V}}\text{CO}}_{\text{2}}$ (Figure 3) and precluded additional experiments.

## Conclusions

The results of the present study demonstrate that ventilating an ECML with up to 300 ppm H_2_S in partial cardiopulmonary bypass circulation does not reduce CO_2 _production or O_2 _consumption in anesthetized sheep. Our results show that diffusion of up to 300 ppm H_2_S into blood via a membrane lung can cause dose-dependent pulmonary vasoconstriction, hypoxemia and catastrophic systemic vasodilation. These observations do not support the hypothesis that administration of a high concentration of H_2_S reduces metabolism in anesthetized large mammals. Whether the administration of H_2_S inhibits metabolism in large mammals when metabolic rate is increased (for example, systemic inflammation or exercise) remains to be determined.

## Key messages

• High concentrations of H_2_S administered via ECML ventilation do not alter CO_2 _production in sheep on partial cardiopulmonary bypass perfusion.

• In this setting, H_2_S poses the risk of pulmonary vasoconstriction, hypoxemia and systemic vasodilation.

• Therefore, administration of high concentrations of H_2_S via membrane lung may not be useful for reducing oxidative metabolism in large mammals.

## Abbreviations

c_a_O_2_: arterial oxygen content; c_e_O_2_: efferent oxygen content; CO: cardiac output; CO_2_: carbon dioxide; c_v_O_2_: mixed venous oxygen content; CVP: central venous pressure; ECML: extracorporeal membrane lung; FeCl_3_: iron(III) chloride; F_E_CO_2_: mean fraction of CO_2 _in expired air; F_i_O_2_: fraction of inspired oxygen; Hb: hemoglobin concentration; HCl: hydrogen chloride; HR: heart rate; H_2_S: hydrogen sulfide; iv: intravenously; MAP: mean arterial pressure; mmHg: millimeters of mercury; MPAP: mean pulmonary artery pressure; NaHS: sodium hydrosulfide; Na_2_S: sodium sulfide; O_2_: oxygen; p_a_CO_2_, PCWP: pulmonary capillary wedge pressure; arterial carbon dioxide tension; pH_a_: arterial pH; ppm: parts per million; pO_2_: oxygen tension; ${\dot{\text{V}}\text{CO}}_{\text{2}}$: carbon dioxide production; ${\dot{\text{V}}\text{O}}_{\text{2}}$: oxygen consumption; ${\dot{\text{V}}}_{\text{E}}$: expiratory minute volume; ${\dot{\text{V}}}_{\text{L}}{\text{CO}}_{\text{2}}$: amount of CO_2 _exhaled from the lungs per unit of time; ${\dot{\text{V}}}_{\text{M}}{\text{CO}}_{\text{2}}$: amount of CO_2 _removed from the circulation via membrane oxygenator per unit of time.

## Competing interests

The authors declare that they have no competing interests.

## Authors' contributions

MD and RCF performed the experiments and data analysis, contributed to the design and interpretation of the study and wrote the manuscript. KK performed plasma H_2_S measurements and helped perform the experiments. MB, EC and CA contributed to the study setup. WMZ and FI contributed to the conceptual design of the study, to the interpretation of data, and to manuscript writing and editing. WMZ and FI contributed equally to this study. All authors have read and approved the final manuscript.
